# Decoding water quality across urban and rural dental clinics: insights from an observational study

**DOI:** 10.3389/froh.2025.1662208

**Published:** 2025-10-30

**Authors:** Bahar Vatanparast, Elkin Florez Salamanca, Amrinderbir Singh, Michelle F. Siqueira

**Affiliations:** University of Saskatchewan College of Dentistry, Saskatoon, SK, Canada

**Keywords:** infection control, water safety, urban-rural, dental clinic, water test

## Abstract

**Introduction:**

Adherence to drinking water standards in dental treatments is a critical measure for preventing nosocomial infections. This study aimed to evaluate water quality from dental unit waterlines (DUWLs) and clinic taps over eight months in urban and rural dental clinics across Saskatchewan, Canada.

**Methods:**

Staff from one urban dental clinic and three rural clinics underwent refresher training on maintaining DUWLs. Training included protocols for flushing lines, using disinfecting tablets, shocking lines with sodium hypochlorite, and proper sample collection. Water samples were aseptically collected from DUWLs and clinic taps using Sigma-Aldrich® waterline test kits and analyzed at a quality assurance laboratory for bacterial contamination. Samples were incubated for seven days and categorized based on bacterial colony counts. Failed DUWL tests (CFU/ml > 500) were repeated after shocking procedures. Statistical analysis included frequency calculations, cross-tabulations, and Chi-square tests, with significance set at *α* = 0.05.

**Results:**

A total of 399 samples were analyzed over eight months. Among DUWL samples, 14.9% from the urban clinic and 36.4% from rural clinics failed quality standards. Tap water from the urban clinic showed no failures, whereas 46.9% of rural tap water samples failed. Urban clinics had faster retesting, with 71% completing retests within one week, compared to 28% in rural clinics. Rural retest failure rates were 33.5% compared to 10% at urban clinics.

**Discussion:**

Disparities in water quality between urban and rural dental clinics in Saskatchewan were evident, with rural clinics exhibiting higher contamination rates and slower remediation actions. These findings underscore the urgent need for enhanced infection control measures, including targeted staff training, implementation of robust waterline maintenance protocols, prompt retesting practices, and consideration of alternative tap water sources in rural settings. Addressing these challenges is essential to ensuring safe and equitable dental care while reducing the risks associated with contaminated water.

## Introduction

Water used during dental procedures is essential for irrigating, cooling, and rinsing oral tissues, with water being delivered through dental units (1, 2). Dental unit waterlines (DUWLs) consist of narrow tubing that transports water from its source, whether municipal water or an external reservoir, to the handpieces, air/water syringes, and ultrasonic scalers (3, 4). During dental procedures, both patients and oral health care providers are regularly exposed to droplets and aerosols generated by dental devices (5, 6). To minimize the risk of nosocomial infections linked to contaminated water, it is essential that water emitted from dental units consistently meets drinking water standards (1, 7, 8).

Procedural water in dental settings can become contaminated through two main mechanisms: the backflow of saliva into dental devices lacking adequate anti-retraction valves and the introduction of microorganisms from the primary water source or storage system (9, 10). Once water enters the DUWLs, the narrow tubing and periods of stagnation when the units are not in use create an optimal environment for microbial growth (3, 11, 12). Infections linked to contaminated DUWLs have underscored the substantial risks posed by poor water quality in dental settings. Numerous outbreaks of respiratory and systemic infections have been traced to biofilms and high microbial counts within DUWLs (6, 13–15).

Two fatal cases of legionellosis in elderly patients highlight the severe risks posed by contaminated DUWLs, particularly to immunocompromised individuals (14, 15). Studies have also shown elevated rates of seropositivity for Legionella antibodies among dental personnel, suggesting heightened exposure risks within dental environments (16, 17). More recently, a facial cutaneous sinus tract infection was linked to *Mycobacterium fortuitum*, *M. abscessus*, and *M. peregrinum* contamination found in DUWLs (18, 19). In addition, documented cases of nontuberculous mycobacterial infections in pediatric patients have led to serious complications, including cervical lymphadenitis (20). These findings underscore the critical importance of implementing stringent DUWL maintenance protocols and routine water quality monitoring to safeguard both patients and oral healthcare providers. Rigorous infection control measures are essential for reducing microbial contamination risks and ensuring the safe delivery of dental care (18–20).

Water contamination levels from DUWLs are measured in Colony Forming Units (CFU) and are based on drinking water standards (21). In Canada, the standard follows the Centers for Disease Control and Prevention's guideline of less than 500 CFU/ml (22). Maintaining high water quality standards in dental practices can be particularly challenging in regions where baseline water quality is already compromised. Many Canadian rural communities are under long-term boil water advisories due to contaminated water (23). As of July 18, 2024, twenty-eight First Nations communities in Canada were under long-term boil water advisories, including three in the province of Saskatchewan, which also had 10 active short-term advisories (24). Part of the challenge stems from the operational limitations of rural clinics, which frequently function with constrained financial resources. Many are situated in areas with outdated water treatment infrastructure and are staffed by personnel who may lack adequate training in critical issues such as water quality, largely due to workforce shortages and high staff turnover (25, 26). When the baseline water quality is already subpar, it exacerbates the challenge of maintaining water quality standards in DUWLs in dental practices, potentially leading to higher risks of waterborne infections for patients (3). Thus, the water security issues in rural communities not only pose direct health risks but also undermine the safety and effectiveness of healthcare services, including dental care (27).

In Saskatchewan, Canada, local regulations mandate annual testing of DUWL water quality (28). However, legislative requirements for DUWL testing vary significantly across regions, creating substantial inconsistencies. These gaps can result in prolonged periods without testing or, in some cases, no testing requirements at all, potentially leaving water quality issues unresolved for extended durations (9). A 2023 retrospective study conducted by our research team evaluated the DUWL water quality of 137 dental clinics in Saskatchewan over an eleven-year period. The study revealed that 21% of DUWL samples failed to meet drinking water standards, emphasizing the ongoing challenge of maintaining water safety in dental practices (29). Regular monitoring and testing of DUWLs are critical to ensuring compliance with safety standards and mitigating risks associated with microbial contamination (30). Despite the clear importance of this issue, there remains a notable lack of research on DUWL water quality, particularly in rural dental clinics. To address these gaps, our study aimed to investigate the quality of both tap water and DUWL water in rural and urban dental clinics across Saskatchewan over an eight-month period, providing a comprehensive assessment of water safety and identifying areas for improvement in infection control practices.

## Materials and methods

The study involved three rural dental clinics and one urban dental clinic in Saskatchewan, Canada. The rural clinics, located within community health centers, each operated two dental units. The urban clinic, based in a teaching institution, housed 85 dental units. All units were equipped with closed water systems featuring independent water reservoirs. A total of 399 water samples were collected from DUWLs. The sample size was selected to ensure meaningful representation across clinic types and to support robust statistical analysis. Practical considerations such as the number of dental chairs and logistical access, particularly in remote areas where amenities like post offices are not always available, were also factored into the sampling strategy.

Clinical staff at all participating sites received in-person refresher training on proper DUWL maintenance protocols. The training reinforced key practices, including the addition of a disinfectant tablet (ICX, A-dec, Newberg, Oregon, USA) to the water reservoir with each refill, flushing DUWLs for two minutes at the start of each day, and for 20–30 s between patients (31). Staff also received hands-on instruction in DUWL shocking procedures using sodium hypochlorite, as well as standardized water sample collection techniques to minimize cross-contamination (31), emphasizing the use of clean gloves and the disinfection of the tap, air-water syringe, handpieces, ultrasonic scaler, and nearby counter surfaces using intermediate-level disinfectant.

Clinics were provided with all necessary materials, including commercial water testing kits (HPC Total Count Sampler, Sigma-Aldrich, Oakville, ON, Canada) for biweekly use, along with pre-paid courier envelopes for sample shipment. Water samples from both taps and DUWLs were collected between October 2023 and May 2024, following the manufacturer's instructions. For each dental unit, DUWL samples were collected as pooled samples, meaning equal volumes of water were drawn from all waterlines (e.g., air-water syringe, handpieces, ultrasonic scaler) within a single unit and combined into one test sample. Importantly, samples from different chairs were not combined; each dental unit was sampled and analyzed individually to maintain data accuracy. All samples were promptly shipped to the University of Saskatchewan's Sterilizer and Waterline Monitoring Service (SWMS) Laboratory for microbiological analysis.

Upon receipt, samples were incubated at room temperature for seven days, and bacterial colonies were subsequently quantified in CFU/ml according to the manufacturer's template. Samples with >500 CFU/ml were considered failed DUWL or tap water tests. Comprehensive reports detailing the findings were generated for each sample and shared with the respective dental clinic staff or other relevant stakeholders. This process ensured timely feedback and actionable insights to maintain water quality and safeguard patient and staff health.

For failed DUWL samples (>500 CFU/ml), the time until retesting was calculated based on the date the subsequent test was received by the SWMS laboratory. Clinics with failed DUWL tests were contacted via email with detailed remediation instructions to shock the system using 0.5% sodium hypochlorite shocking solution for 10 min (9, 31). If tap water samples failed, community stakeholders were informed, as the tap water could potentially impact other community areas.

Absolute and relative frequencies were calculated to summarize the findings. Crosstabs were used to determine observed frequencies, and a Chi-square test was conducted to assess statistically significant differences between test results from urban and rural clinics. The analysis was performed using IBM® SPSS® Statistics version 28.0 (IBM Corp., Armonk, N.Y., USA) with a significance level of 0.05.

## Results

**Figure 1:**
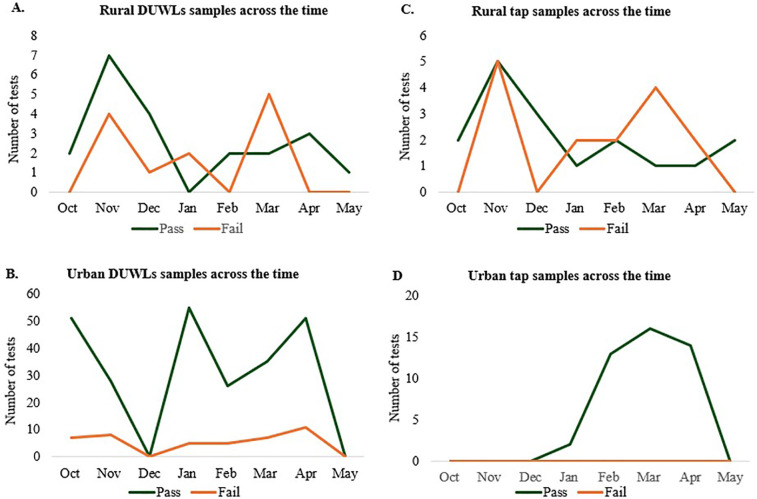
Overall clinic participation during the study period. Number of DUWL water samples tested across the study in **(A)** rural clinics and **(B)** urban clinic. Number of tap water samples tested across the study in **(C)** rural clinics and **(D)** urban clinic.

A total of 399 water samples were collected during the study period, including 334 from the urban clinic and 65 from rural clinics. Sampling frequencies for DUWLs and tap water varied over time (Figure 1). Of these, 80.7% were DUWL samples, and 19.3% were tap water samples. Overall, 80.5% of tap water samples and 82.9% of DUWL samples were found to be suitable for human consumption (<500 CFU/ml).

**Figure 2:**
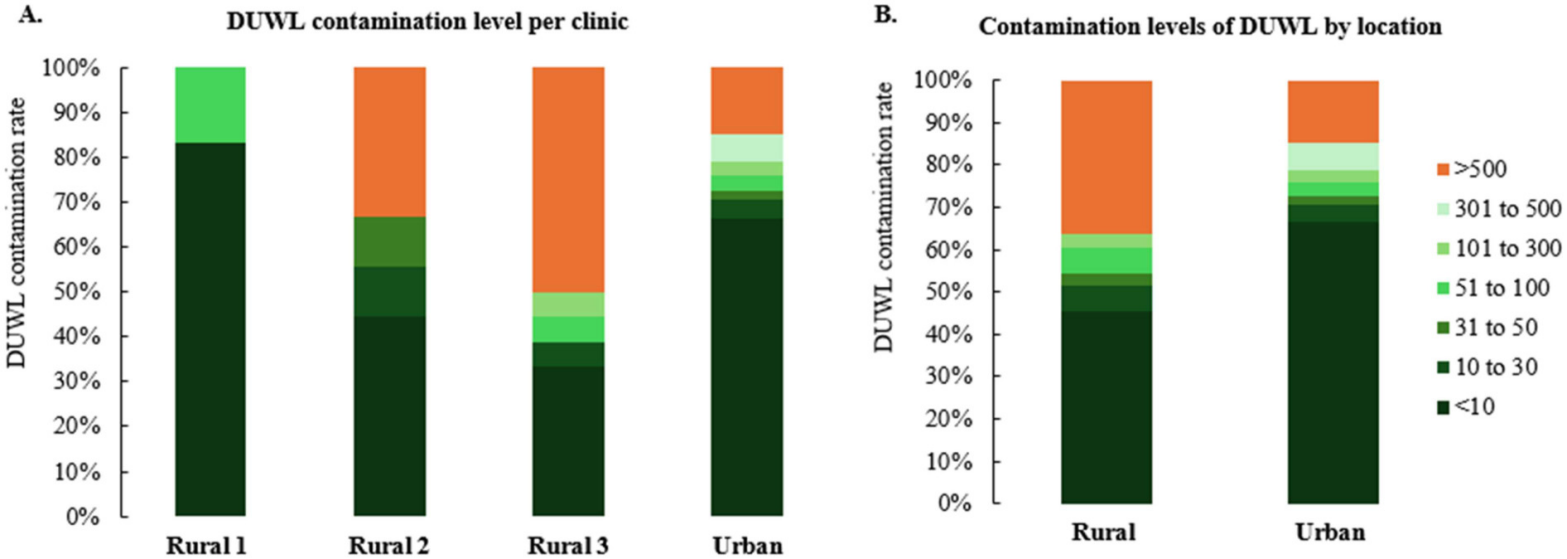
Contamination levels observed in water samples from DUWLs during the study period. The data are presented by dental clinic denominations **(A)** and location **(B)** variables.

**Figure 3:**
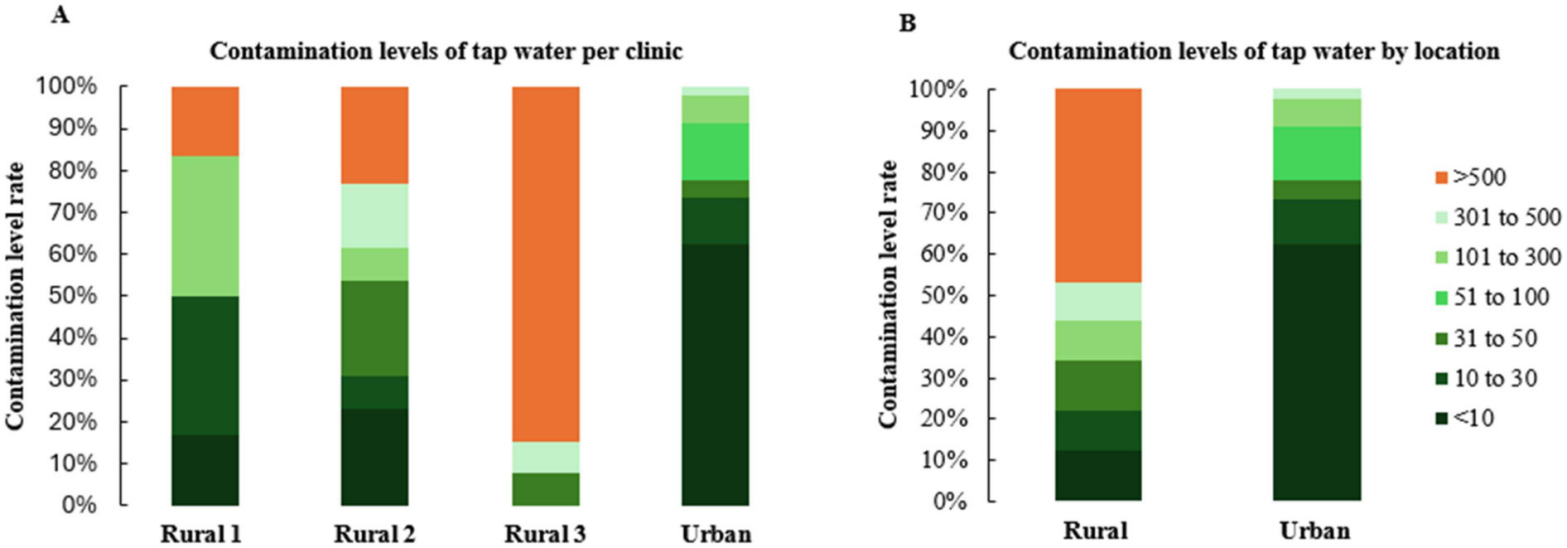
Water contamination levels observed in tap water samples during the study period. The data are presented by dental clinic denominations **(A)** and location **(B)** variables.

For DUWL samples, the urban clinic reported a failure rate of 14.9% (95% CI: 11.2–19.4%, *n* = 289), while rural clinics exhibited failure rates ranging from 0%–50% (Figure 2A), with an overall rural failure rate of 36.4% (95% CI: 22.2–53.4%, *n* = 33). Rural clinic's failure rate was significantly higher than the urban clinic's failure rate (*p* = 0.02) (Figure 2B). Tap water failures showed considerable variability among rural clinics, with rates ranging from 16.7%–84.6% (Figure 3A), and an overall rural tap water failure rate of 46.9% (95% CI: 30.9–63.6%, *n* = 32). In contrast, no tap water failures were observed in the urban clinic (Figure 3B).

**Figure 4:**
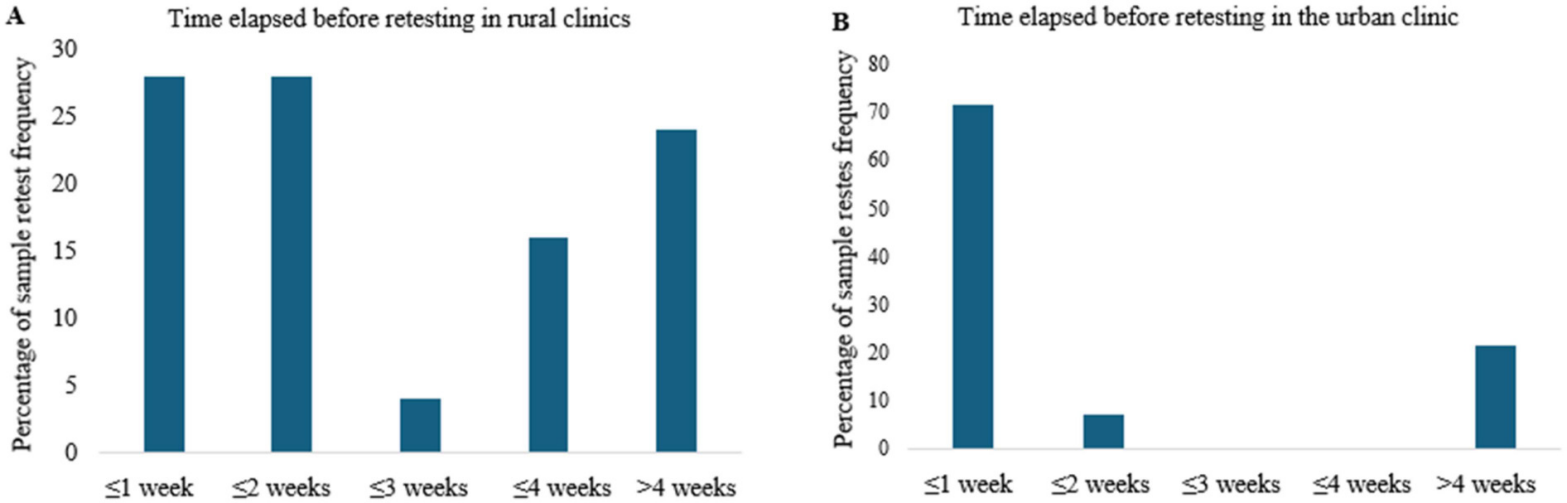
The time elapsed before retesting failed DUWL and tap water samples was recorded for both rural **(A)** and urban **(B)** clinics. This elapsed time was calculated by measuring the interval between the date the clinic was notified of a failed test and the date the subsequent test was performed on the same DUWL or tap water source.

**Figure 5:**
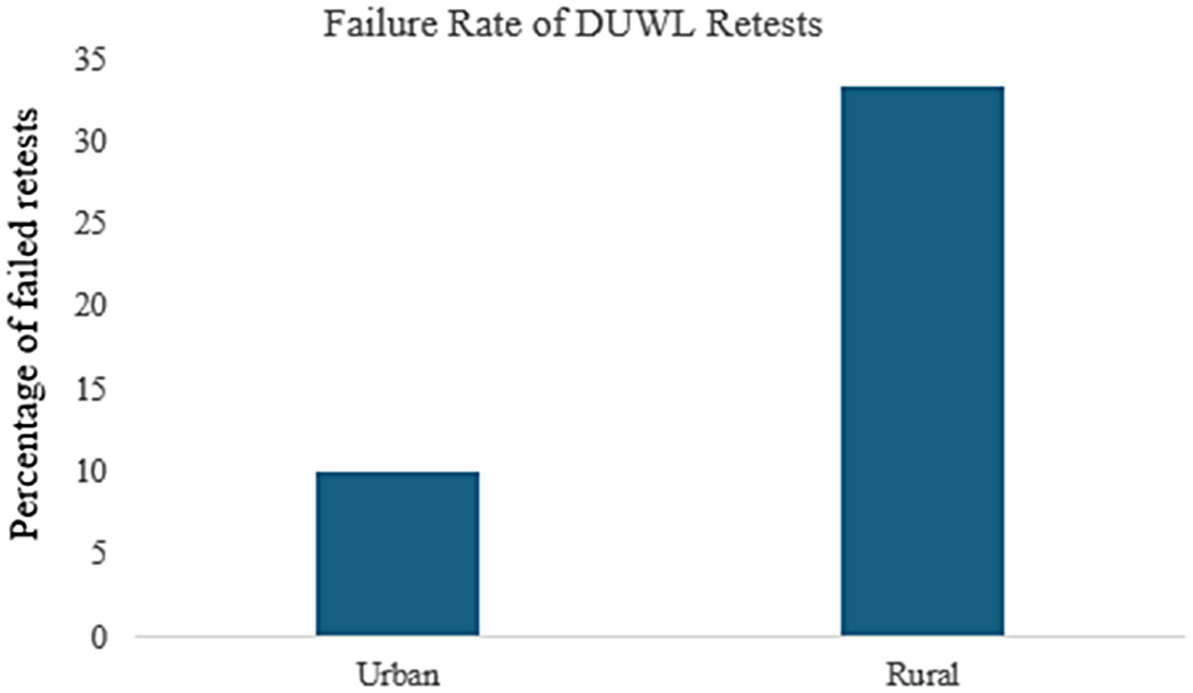
DUWL retest failure rate in urban vs. rural clinics: Tests were classified as retests if the preceding test on the same DUWL had resulted in a failure.

Retesting following failed DUWL tests also showed differences between urban and rural clinics. In the urban clinic, 71% of failed DUWL tests were retested within one week of notification, compared to only 28% in rural clinics (Figure 4). Of the retested DUWL samples, 10% in the urban clinic failed again (95% CI: 4.0–23.1%, *n* = 40), whereas 33.5% of retested samples in rural clinics resulted in repeat failures (95% CI: 12.1–64.6%, *n* = 9) (Figure 5).

## Discussion

In dentistry, minimizing the risk of pathogen exposure and cross-infection during dental procedures is crucial (32). The failure rate of DUWLs in the urban clinic of this study, at 14.9%, reflects an improvement compared to our previous research, which reported a 21% failure rate for DUWLs in Saskatchewan over an eleven-year period (29). However, the significantly higher failure rate in rural clinics (36.4%) underscores the greater challenges associated with maintaining water quality in these areas.

Contamination levels in DUWLs and tap water at the urban clinic were lower than those reported in other studies. For instance, an Italian study found that 58.8% of DUWL samples from seven teaching hospitals and three non-teaching hospitals exceeded the recommended 500 CFU/ml (33). Similarly, a study from the Dental College in Mashhad, Iran, reported an initial contamination rate of 36.1% with *Legionella pneumophila*, which decreased to 5.7% following a 2 min turbine flushing (34).

Our study revealed a notable disparity in bacterial contamination between urban and rural clinics. Rural clinics exhibited higher contamination rates, with 36.4% of DUWLs and 46.9% of tap water samples failing to meet drinking water standards. One rural clinic, in particular, had an alarming failure rate, with 84.6% of tap water and 50% of DUWL samples exceeding acceptable bacterial limits. This aligns with previous research indicating poor water quality in some rural communities, where 56%–67% of household tap water samples may exceed microbial contamination safety limits (35). Rural populations may face elevated risks due to limited infrastructure, reduced access to routine maintenance, and logistical barriers that delay sample processing and equipment servicing. These challenges can contribute to persistent microbial contamination, posing a greater threat to vulnerable groups such as children and older adults, who are more susceptible to waterborne infections due to age-related or immunological factors. In contrast, urban clinics showed a lower DUWL failure rate of 14.9% and no failures in tap water samples. This lower rate may be attributed to better infrastructure and access to water that meets drinking water standards in urban areas (36). We hypothesize that the elevated DUWL failure rates observed in rural clinics may be partially attributable to insufficient awareness of local water quality conditions. In many rural communities, persistent boil water advisories and limited water treatment infrastructure contribute to the use of untreated or microbiologically compromised tap water. There appears to be a lack of understanding among some clinical staff that such water is unsuitable for use in dental procedures. In certain cases, the absence of explicit advisories may be misinterpreted as an indication that the water is safe, potentially leading to the inadvertent use of contaminated water during patient care. These results highlight the challenges faced by many rural communities, including limited access to advanced water treatment facilities. From 2010–2021, most boil water advisories in Canada were issued in rural communities with populations of 500 or fewer, primarily due to operational challenges (35). For locations experiencing ongoing water quality challenges, dental clinics could consider engineering solutions, like isolating dental devices from municipal water, to enhance safety (37, 38). Persistent urban–rural disparities underscore the urgent need for systemic reforms in oral healthcare delivery. Rural communities often operate with fewer financial resources, limited access to specialized and modern technology, and face significant shortages of oral healthcare providers. These structural limitations contribute to and intensify oral health inequities commonly observed in rural populations. Addressing these disparities requires culturally sensitive, community-led strategies that not only improve oral health outcomes but also promote equity in access, service quality, and overall health status among underserved rural populations. Additionally, because water testing services are typically centralized in urban areas, rural clinics often face significant logistical barriers to timely and consistent monitoring. Many of these clinics are located at a considerable distance from postal service facilities, and in some cases, rely on infrequent or unreliable mail delivery. These factors can lead to delays in shipping DUWL samples, ultimately postponing necessary remediation steps. For instance, in this study, delays in the arrival of retest samples from rural clinics resulted in prolonged reporting times. Compounding these logistical issues, one-third of rural DUWL samples continued to exceed acceptable microbial limits even after retesting. These persistent challenges may also reflect high staff turnover in rural clinics, which can limit opportunities for comprehensive training and reduce adherence to standardized infection control protocols.

Existing studies provide clear guidelines for monitoring, treating, and maintaining DUWLs (29–31), but the implementation of these practices often relies on healthcare professionals who may lack formal training in DUWL care. Moreover, demographic and socioeconomic factors may also be contributing to a lack of awareness about the risks associated with using contaminated tap water for consumption and in healthcare settings in rural areas (39, 40). To address these challenges, implementing additional training and establishing in-clinic routines for regularly scheduled disinfection procedures could be highly beneficial. The use of alternative water sources, such as distilled or bottled water, may also help improve water quality outcomes. Notably, the urban clinic was situated within a learning institution, where students were consistently supervised by experts, potentially explaining the lower failure rates compared to other studies. Future research should include private dental clinics in urban areas to gain a more comprehensive understanding of these issues across diverse settings. Current regulations in Saskatchewan require only annual waterline testing, but our study showed that numerous dental units failed water quality tests during an eight-month period. Annual testing could thus pose a risk due to lengthy periods of unrecognized contamination and potential health hazards for both patients and dental staff (28). Regular and more frequent testing is essential to ensure water safety and to protect public health.

## Limitations

This study was conducted in a limited number of dental clinics, all of which are affiliated with a single teaching institution. While this facilitated consistency in operational standards and data collection procedures, it may introduce institutional bias and limit the generalizability of findings to clinics with different management structures or operational protocols. Additionally, the small sample size, though purposeful for feasibility, restricts the breadth of representation across the province. However, the rural clinics included in the study were selected for their similarity to many other community-based dental practices in remote areas of Saskatchewan, supporting the relevance of the findings to similar settings. Future studies involving a broader range of clinic types and management models would help validate and expand upon these results. Additional limitations should be considered when interpreting the findings of this study. First, the use of pooled sampling may obscure which specific waterline within a dental unit was contaminated. This approach, while practical, may limit the detection of localized contamination. Second, variability in staff training and adherence to waterline maintenance protocols may have influenced sample integrity. Although all clinics followed institutional guidelines, differences in implementation and staff turnover could affect consistency in maintenance practices and sampling procedures. Third, logistical challenges in rural areas, such as limited access to postal services, occasionally delayed sample shipment and processing, potentially impacting microbial viability and detection. These factors may introduce bias and should be considered when generalizing the results to other dental settings. Furthermore, in the statistical analyses, multiple samples were collected from the same clinics, resulting in non-independent observations. Consequently, the calculated confidence intervals may underestimate the true variability in water quality outcomes, and this limitation should be considered when interpreting the findings. Additionally, given the limited number of participating clinics (*n* = 4), regression analysis to adjust for clinic-level effects was not conducted. With such a small number of sites, statistical adjustments for clinic-level effects would not yield reliable or interpretable estimates and could compromise the validity of the results. As a result, unmeasured differences at the clinic level may have influenced the findings and should also be considered when interpreting the results.

## Conclusion

The DUWL and tap water failure rates observed over this eight-month study underscore the urgent need for broader, long-term investigations to determine whether these patterns are prevalent across other regions. Further exploration is also needed to understand the underlying causes of these geographic disparities, especially considering the serious implications of water contamination for high-risk populations and the persistent water security issues often faced by remote and rural communities. Based on the findings in this study, regulatory bodies should consider revisiting the current requirements for water quality monitoring, particularly the mandated frequency of testing, and implement stronger mechanisms to enforce compliance with DUWL safety standards. Government agencies must also prioritize infrastructure improvements in underserved areas and ensure that dental clinics are equipped to deliver safe care. Equally important is the need to strengthen the education of oral health care providers regarding DUWL maintenance. This includes enhancing awareness of water quality risks, proper daily maintenance protocols, frequent shocking procedures, and routine testing. Incorporating these topics into stricter continuing education requirements focused on infection prevention and control could significantly improve provider knowledge and practice. From a broader public health perspective, these findings highlight the critical need to integrate DUWL safety into broader water quality and infection control policies. Failure to address these issues may contribute to preventable disease transmission, particularly among immunocompromised individuals and other vulnerable groups. A sustained effort to embed waterline care into both initial training and ongoing professional development is essential to ensuring safe dental care environments across all settings. Ultimately, these insights should inform public health policy and resource allocation, guiding targeted interventions that reduce health disparities and improve water safety standards in clinical settings.

## Data Availability

The raw data supporting the conclusions of this article will be made available by the authors, without undue reservation.
